# Communicating health risk in chronic kidney disease: a scoping review

**DOI:** 10.1007/s40620-024-02098-0

**Published:** 2024-11-08

**Authors:** Emma Caton, Ros Aird, Maria Da Silva-Gane, Sivakumar Sridharan, David Wellsted, Shivani Sharma, Ken Farrington

**Affiliations:** 1 School of Life and Medical Sciences, University of Hertfordshire, Hertfordshire, UK; 2 Lister Area Kidney Patients Association, Stevenage, UK; 3 Department of Renal Medicine, Lister Hospital, East and North Hertfordshire NHS Trust, Stevenage, UK; 4 College of Business and Social Sciences, Aston University, Birmingham, UK

**Keywords:** Risk communication, Chronic kidney disease, Shared decision-making, Prognosis

## Abstract

**Background:**

Communicating risk is a key component of shared decision-making and is vital for the management of advanced chronic kidney disease (CKD). Despite this, there is little evidence to suggest how best to communicate health risk information to people living with CKD. The aim of this review was to identify and understand the nature of evidence-based risk communication strategies for people living with CKD.

**Methods:**

We searched MEDLINE, CINAHL and Scopus databases for articles which described or evaluated the use of risk communication strategies within the renal population. Similar risk communication strategies were collated and summarised narratively.

**Results:**

A total of 3700 sources were retrieved from the search, of which 19 were included in the review. Eleven studies reported primary research, and eight reported either narrative or systematic reviews. Seven main risk communication strategies were identified: framing, absolute versus relative risk, natural frequencies versus percentages, personalised risk estimates, qualitative risk communication, best-case/worst-case framework and use of graphs and graphics. There was a paucity of risk communication strategies specific to the CKD population.

**Conclusion:**

Evidence-based strategies to improve health risk communication for patients living with CKD are lacking. There is a need to establish the informational and communication preferences for patients living with CKD to better understand how to best communicate health risk information to individuals in this population.

**Graphical abstract:**

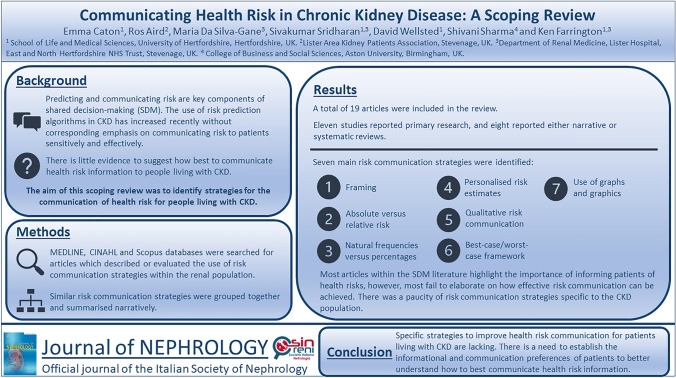

**Supplementary Information:**

The online version contains supplementary material available at 10.1007/s40620-024-02098-0.

## Introduction

Shared decision-making describes a process in which patients work together with healthcare professionals to make decisions about their care. In shared decision-making, treatment options are chosen based on a combination of scientific evidence and the patient’s individual preferences, goals and values [1]. Shared decision-making is a core principle of medical practice and has been associated with positive outcomes such as greater patient satisfaction, increased motivation to adherence to treatment, improved quality of life, and reduced decisional conflict [2, 3].

A key component of shared decision-making is the communication of risk. Communicating risk to patients with life-limiting long-term conditions is a major issue. There have been significant advances in predicting risk in patients with long-term conditions, and there are increasing numbers of risk-predicting algorithms. Despite this, there is little research on how to apply these tools in clinical practice. For example, European Best Practice Guidelines for management of advanced chronic kidney disease (CKD) in older adults [4] recommends using three different risk prediction models together to help healthcare professionals and people living with CKD to estimate the risk of disease progression, of dying before end-stage kidney disease and the need for dialysis, and their likely prognosis should they start dialysis. This information is vital for making the appropriate decisions about clinical management in terms of planning for renal replacement therapy, conservative management, or end-of-life planning. However, there is little work guiding the use of such tools which relate to highly sensitive issues affecting very vulnerable patients and their carers.

Many patients would like to receive information about their prognosis, however healthcare professionals are often reluctant to disclose this information [5, 6]. Some healthcare professionals have expressed concerns about diminishing hope, providing uncertain information or lacking the skills necessary for prognosis communication [5, 7]. Potential harms of not disclosing risk information include the establishment of unrealistic expectations in the minds of patients and their families and carers. In a study conducted by Ghanem et al., 77% of patients were found to be in prognostic discordance with their nephrologist, suggesting that most patients overestimate their chance of survival [8]. This highlights the importance of shared decision-making and effective risk communication in renal care, as patients who overestimate their prognosis may opt for more intensive or invasive treatment options perhaps with little prospect of benefit [8]. Despite this, evidence suggests that shared decision-making is not routinely adopted in renal care. For example, in a study conducted by Frazier et al., less than half of older adults with advanced CKD agreed that their decision about treatment was made in collaboration with their doctor [9].

The aim of this scoping review was to identify and understand the nature of evidence-based risk communication strategies for people living with CKD. A scoping review methodology was considered suitable to understand how risk communication has been conceptualised and implemented within renal decision-making, and identify the breadth of the literature.

## Methods

A study protocol was established to help guide the review process. This review was not pre-registered with PROSPERO as scoping reviews are ineligible for registration on this database.

### Search strategy

A literature search was conducted on 28th March, 2023. Studies were identified using MEDLINE, CINAHL and Scopus databases. Additional sources were identified from the secondary research articles included in the review (e.g. where a specific risk communication strategy had been referenced, the original source was obtained). Search terms were generated around concepts related to kidney disease (e.g. “kidney disease”, “dialysis”, “conservative management”) and risk communication (e.g. “risk communication”, “prognosis communication”, “shared decision making”). No date limit was applied to the search. The full search strategy can be found in Table S1 (See Supplementary information).

### Study selection and data extraction

To be eligible for inclusion, studies needed to describe or evaluate the use of health risk communication strategies within the renal population. With the exception of case studies, case series, and case reports, all study designs were eligible for inclusion in the review. Due to the limited language resources available to the research team, studies were only included in the review if they had been published in the English language. Where the full-text version of a manuscript could not be obtained, the article was excluded from the review. Articles were also excluded if they exclusively described risk communication strategies aimed at caregivers, surrogate decision-makers or paediatric patients, or if they addressed the risks associated with COVID-19.

Search results were extracted into the reference management tool Rayyan [10]. Duplicate articles were identified by the tool and then manually removed by the researcher. Articles were initially screened via their title and abstract, with ineligible articles being excluded from the review. The remaining articles underwent screening via their full text. A subsection of full-text articles (18%) were independently assessed by two authors (RA and MDSG) and any conflicts were discussed and resolved within the research team.

Key data were extracted from each source. The data extracted included study characteristics (e.g. study title, study authors, year of publication, study design, study population), a description of the risk communication strategies, and key findings such as the impact of these strategies on patient’s knowledge, perception of risk and/or treatment decisions.

### Synthesis of results

Similar risk communication strategies were grouped together and summarised narratively. Both primary and secondary research studies have been included. Due to the heterogeneity of study methods, a risk of bias (quality) assessment was not performed.

## Results

### Study selection

**Fig. 1:**
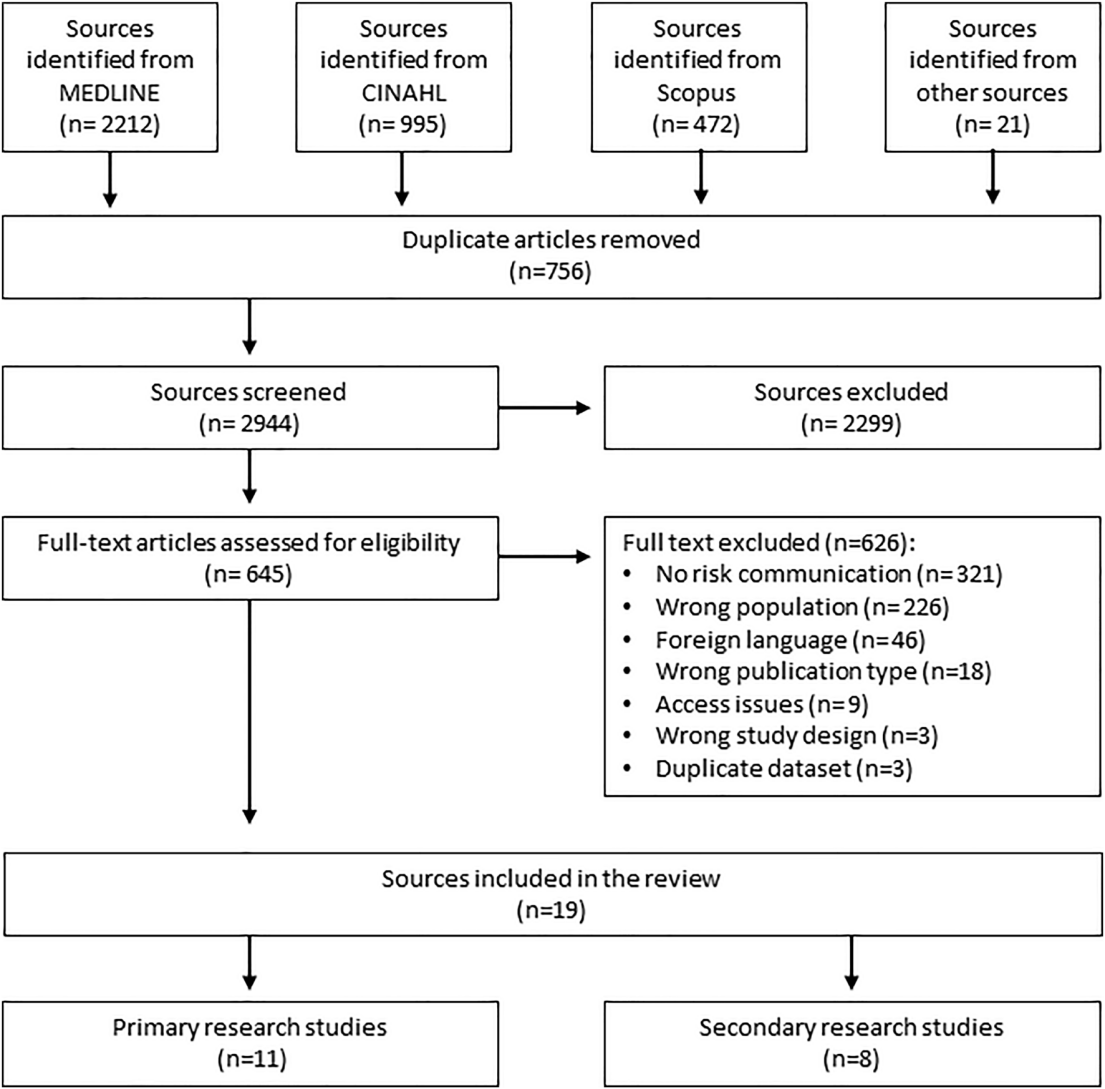
Study selection PRISMA flow diagram

A total of 3700 sources were identified. A PRISMA flow diagram outlining the full study selection process can be found in Fig. 1. Full-text screening was performed on 645 articles. Overall, a total of 19 studies were identified for inclusion in the review.

### Study characteristics

Eleven studies reported primary research (see Table 1). This included three qualitative studies[11–13], three mixed method studies [14–16], two intervention studies [17, 18], one survey study [19], one validation study [20]and one discrete choice experiment [21]. The intervention studies evaluated the use of the iChoose Kidney patient decision aid [17] and the best-case/worst-case communication tool [18]. Overall, six studies described the development, evaluation or use of patient decision aids, risk prediction models or other tools to support the shared decision-making process [13, 15–18, 20]. Six studies included patients [14, 16, 17, 19–21], four included patients and healthcare professionals [12, 13, 15, 18] and one study included only healthcare professionals [11].

**Table 1:** Characteristics of primary research studies

Study ID	Year of publication	Country	Study design	Type/number of participants	Risk communication strategies/components	Evaluated the impact of risk communication strategies?
Cardinal et al. [11]	2020	Canada	Qualitative interview study	HCPs (*n* = 15)	Personalised risk estimates Use of graphs and graphics Interpersonal communication skills Managing uncertainty Shared decision-making tools and patient education	No
Damron et al. [19]	2022	USA	Survey study	Patients (*n* = 1029)	Natural frequencies versus percentages	No
Dowen et al. [14]	2017	New Zealand	Mixed methods survey study	Patients (*n* = 177)	Use of graphs and graphics	Yes
Engels et al. [15]	2022	Netherlands	Mixed methods study (Focus groups and survey)	Patients (*n* = 133) and HCPs(*n* = 51)	Use of graphs and graphics Shared decision-making tools and patient education	No
Finlay et al. [12]	2020	Canada	Qualitative interview study	Patients (*n* = 20) and HCPs (*n* = 10)	Personalised risk estimates Qualitative risk communication Shared decision-making tools and patient education	No
Ozdemir et al. [16]	2021	Singapore	Mixed methods study (Interviews and survey)	Patients (*n* = 20) and caregivers (*n* = 12)	Use of graphs and graphics Shared decision-making tools and patient education	No
Patzer et al. [17]	2018	USA	Intervention study (Randomised controlled trial)	Patients (*n* = 470)	Absolute versus relative risk Use of graphs and graphics Shared decision-making tools and patient education	Yes
Peeters et al. [20]	2016	Belgium	Validation study	Patients (*n* = 3472; registry data)	Use of graphs and graphics Shared decision-making tools and patient education	No
Tuot et al. [13]	2022	USA	Qualitative think-aloud study	Patients (*n* = 18) and HCPs (*n* = 19)	Interpersonal communication skills Shared decision-making tools and patient education	No
Wilson et al. [21]	2023	Canada	Discrete Choice Experiment	Patients (*n* = 140)	Use of graphs and graphics	No
Zimmermann et al. [18]	2020	USA	(Pre/post-) Intervention study	Patients (*n* = 30) and HCPs (*n* = 16)	Best-case/worst-case framework Shared decision-making tools and patient education	Yes

HCPs = Health care professionals

Eight studies were secondary research studies, including seven narrative reviews and opinion pieces [22–28]and one systematic review [29]. The systematic review aimed to evaluate patient decision aids for people with advanced kidney disease. Seventeen patient decision aids were identified in the review. None of the decision aids were eligible for inclusion in the current review due to the lack of published data or insufficient reporting of risk communication strategies.

### Risk communication strategies

Seven main risk communication strategies were identified from the literature and have been summarised in Table 2.

**Table 2:** Risk communication strategies

Risk communication strategy	Description of risk communication strategy	Articles including the risk communication strategy
Framing	Presenting information using either positive or negative terms (i.e. chance of survival versus chance of death)	[25, 29]
Absolute versus relative risk	Presenting the actual likelihood of an event occurring (absolute risk) versus the likelihood of an event occurring in one group compared to another group (relative risk)	[17, 24, 27]
Natural frequencies versus percentages	Presenting information using natural frequencies (e.g. 1 in 5) versus percentages (e.g. 10%)	[19, 29]
Personalised risk estimates	Calculating a person’s individual risk using multiple predictors specific to the individual	[11, 12]
Qualitative risk communication	Presenting information using words (e.g. high risk, low risk) rather than numbers	[12, 26]
Best-case/worst-case framework	A visual aid which depicts two treatment options. Clinicians use narrative storytelling to describe the “best”, “worst” and “most likely” scenarios for each option	[18, 23]
Use of graphs and graphics	Presenting information using graphs or pictures	[11, 14–17, 20, 21, 28, 29]

### Evaluation of risk communication strategies

#### Framing

Two studies described framing as a useful strategy to communicate health risk [25, 29]. Scherer et al. [25] recommend that risk information be presented in a way that is balanced (i.e. focussing on both the positive and negative outcomes). Winterbottom et al. [29] identified two patient decision aids which used positive and negative frames to communicate health risk information, although the impact of this technique on patient outcomes was not reported.

#### Absolute versus relative risk

Absolute and relative risk estimates were mentioned in three studies [17, 24, 27]. Cassidy et al. [27] suggested that relative risk estimates may be easier to understand than absolute risk, although limited empirical evidence was presented. Patzer et al., [17] described the use of absolute and relative risk estimates to present health risk information within the iChoose Kidney decision aid. Findings suggest that the change in patients’ knowledge of absolute and relative mortality risks pre- to post- intervention was significantly higher among patients receiving the iChoose Kidney intervention compared to those receiving standard education (control). There was no significant difference between the intervention and control group in relation to decisional conflict or treatment preference.

#### Natural frequencies versus percentages

Damron et al. [19] speculated that patients may interpret risk information differently depending on whether risk is presented as percentages or natural frequencies. Winterbottom et al. [29] identified three patient decision aids which presented risk information in the form of percentages, and five which reported natural frequencies with either the same (*n* = 4) or different denominators (*n* = 1). The impact of these techniques on patient knowledge, risk perception or treatment decisions was not evaluated.

#### Personalised risk estimates

Two studies mentioned personalised risk estimates [11, 12]. In both studies, healthcare professionals reported that being able to personalise risk scores using key patient characteristics would help to support the shared decision-making process by making information more relevant to patients. None of the studies evaluated patient preference for how personalised risk data should be presented, nor did the studies examine the impact of this technique on patient knowledge, understanding, or treatment decision-making.

#### Qualitative risk communication

Two studies reported the use of qualitative methods to convey risk [12, 26]. For example, a review of patient information leaflets for living donor kidney transplantation found that 35% of leaflets presented risk qualitatively [26]. Similarly, in a study conducted by Finlay et al. [12], clinicians presented the risk of needing dialysis following a coronary procedure as “high, medium or low risk.” The use of qualitative methods was perceived by clinicians as useful in reducing the density of information presented to patients, although patient preference for this technique was not evaluated.

#### Best-case/worst-case framework

Two articles highlighted the “Best-case/Worst-case” framework as a useful method for discussing risk and promoting shared decision-making [18, 23]. The “Best-case/Worst-case” tool is primarily a visual aid depicting two treatment options. Clinicians use narrative storytelling to describe the “best”, “worst” and “most likely” scenarios for each option. This allows clinicians to incorporate their knowledge of the risk and benefits of each treatment option whilst also prioritising the concerns and values of the patient. In a pilot study conducted by Zimmermann et al., [18] the “Best-case/Worst-case” tool was used to improve shared-decision making about dialysis initiation in older adults with life-limiting kidney disease. The study found that use of the “Best-case-Worst-case” tool can positively influence treatment decisions, with patients of nephrologists who used the tool being less likely to initiate dialysis and more likely to be referred to palliative care. As well as being used for decisions about dialysis initiation, Highet et al. [23] recommend that the “Best-case-Worst-case” tool also be used by transplant providers to aid decisions surrounding transplantation and high-risk donor organs.

#### Use of graphs and graphics

The use of visual aids, such as illustrations or graphs, was mentioned in nine studies [11, 14–17, 20, 21, 28, 29]. Pictographs were frequently used in patient decision aids to communicate health risk [15–17, 20], as well as being incorporated within research materials such as discrete choice surveys [21, 28]. The use of pictographs as a risk communication strategy was not evaluated in these studies. Nevertheless, in a study conducted by Cardinal et al. [11], nephrologists acknowledged that the use of pictures could help transplant candidates to better understand statistical information related to graft- and patient- survival.

Graphs can often convey more information to patients compared to statistical data alone. One study investigated comprehension and patient preference for different graphs in people with chronic kidney disease [14]. Most participants were able to correctly interpret Kaplan Meier curves, pie charts, histograms and pictograms, and 87% of participants found graphs useful in aiding their understanding. Participants mentioned that clear, simple visual aids were particularly useful for CKD risk communication, as their interpretation of complex information may be affected by their condition (“when you have kidney failure the brain is slower”).

### Important components of health risk communication

#### Interpersonal communication skills

The practical components of risk communication were mentioned in several studies. These mostly focused on the interpersonal skills required by healthcare professionals when disclosing prognosis information to patients with CKD.

Prior to any discussion with patients about prognosis information, clinicians should establish how much the patient already knows about their condition and/or treatment options, and how much information they would like to receive [22]. Clinicians should recognise the emotional impact that prognosis discussions can have on the patient, acknowledge their emotions, and respond with empathy [22, 25].

Where possible, the use of ambiguous language and clinical/statistical jargon should be avoided [13, 22]. In a study conducted by Tout et al. [13], clinicians identified potential tension between lay and medical terminology for CKD. Clinicians expressed concerns that in some cases, patients may not recognise the term “Chronic Kidney Disease” because clinicians are more likely to use descriptive terms such as “your kidneys are not functioning properly” rather than naming the condition directly. The use of consistent terminology and risk communication strategies between clinicians is also important in improving patients’ understanding of risk. In a study conducted by Cardinal et al., [11], transplant nurses reported that the type of information provided to transplant candidates often varied between transplant nurses.

#### Managing uncertainty

Three studies mentioned that clinicians should discuss the uncertainty and reliability of risk estimates with their patients [11, 22, 25]. It is important to acknowledge that outcomes such as survival and disease progression can be hard to predict, and that estimates derived from large populations can make it difficult to provide a precise estimate for individual patients [11]. Some articles recommend managing uncertainty by avoiding exact time frames, for example, by using ranges (e.g. “hours to days”, “days to weeks”, “weeks to months”) instead of specific dates or percentages [22].

#### Shared decision-making tools and patient education

The use of decision aids and patient education programmes were identified by clinicians as key facilitators of shared decision-making [11, 12]. In Cardinal et al. [11], nephrologists reported that it was easier to present transplant candidates with information about deceased donor organs when candidates had previously received education about the different types of deceased donors. Whilst clinicians may deem educational opportunities as beneficial to the shared decision-making process, patients noted that CKD education could be improved by including concrete actionable recommendations to reduce the risk of disease progression [13].

In this review, six studies reported the development, evaluation or use of shared decision-making tools such as patient decision aids or risk prediction models [13, 15–18, 20]. A further 17 patient decision aids for CKD have been identified in the systematic review conducted by Winterbottom et al. [29]. Findings from these studies suggest that shared decision-making tools can improve patient knowledge and understanding of kidney disease and its treatments, influence treatment decisions, and reduce decisional conflict.

## Discussion

The aim of this scoping review was to identify strategies for the communication of health risk for people living with CKD. The majority of articles within the shared decision-making literature highlight the importance of informing patients of the risk and benefits of each treatment option, however, most fail to elaborate on how effective risk communication can be achieved.

Findings from this review suggest that there are very few evidence-based risk communication strategies specific to the CKD population. Less than 30% of the primary research articles identified in this review evaluated the impact of risk communication strategies on patient outcomes. Indeed, this creates challenges in assessing the acceptability and effectiveness of risk communication strategies within the CKD population. In the primary research articles that did evaluate the impact of risk communication strategies, the main outcomes which were assessed were patient knowledge, information preferences, decisional conflict, and treatment decisions. Improvement in outcomes such as patient knowledge, decisional conflict and decision regret would demonstrate the effectiveness of risk communication strategies. The use of patient reported outcome measures can be useful tools for evaluating the impact of risk communication strategies and should be utilised more frequently within shared decision-making research.

One potentially useful risk communication strategy identified in this review is the use of personalised risk estimates. In renal medicine, several risk prediction models have been developed to obtain personalised risk estimates relating to disease progression and mortality [30]. Whilst these estimates are important for determining the clinical management of a patient, it is also important to consider other prognostic factors which may be of value to patients. For example, in a meta-synthesis of qualitative studies, patients stated that they based their treatment decisions on which modality would be least intrusive in their lives [31]. This suggests that effective risk communication may involve more than merely presenting the risk of clinical outcomes. In addition, data relating to the use of such prognostic tools in individual consultations with patients is sparse.

Several studies noted that patient preferences for risk information should be considered throughout the shared decision-making process, especially when determining the amount and type of information a patient would like to receive. Clinicians may require additional time to build a rapport with their patients and understand their informational preferences before disclosing prognostic information. This is particularly pertinent in contexts where factors such as language and/or literacy may complicate communication further. Future research involving people with CKD is needed to identify the factors important to patients during discussions about risk, to understand patient preferences regarding the type of information they would like to receive, and to explore effective strategies for risk communication, acknowledging that patients may have unique needs depending on life and social factors.

There are several limitations of this review. First is the strict focus on communication strategies for disclosing risk information, which may have resulted in articles which describe more general communication strategies for kidney-related shared decision-making to be excluded from the review. Similarly, this criterion may have resulted in the exclusion of several CKD-specific decision aids or risk algorithms which did not explicitly describe how risk information was presented. Nevertheless, it is important to differentiate patient decision aids and risk prediction models as tools to support the communication of health risk, rather than being risk communication strategies in and of themselves. Second, this review did not include foreign language articles or unpublished (grey) literature due to limited time and practical resources. There are also limitations which are more broadly associated with scoping review methodology. For example, scoping reviews aim to provide an overview of existing literature in order to identify potential areas for future research [32]. As a result, scoping reviews do not assess the quality of included studies nor do they make judgements as to the ‘weight’ of evidence associated with particular interventions [33]. In spite of this, a scoping review methodology was deemed appropriate to meet the aims of this review, especially given the lack of current knowledge of risk communication strategies within the CKD population.

Overall, findings from this review suggest that specific strategies to improve health risk communication for patients living with CKD are lacking. Further research is needed to explore the informational and communication preferences for patients living with CKD in order to better understand how risk can be communicated effectively within the renal setting.

## Supplementary Information

Below is the link to the electronic supplementary material.

Supplementary file1 (PDF 141 kb)

## Data Availability

All relevant data are included in this published article.

## References

[1] National Institute for Health and Care Excellence (2021) Shared decision making NICE guideline 34339147

[2] Amir N, McCarthy HJ, Tong A (2021) A working partnership: a review of shared decision-making in nephrology. Nephrology 26:851–857 34010487 10.1111/nep.13902

[3] Aubree Shay L, Lafata JE (2015) Where is the evidence? A systematic review of shared decision making and patient outcomes. Med Decis Mak 35:114–131 10.1177/0272989X14551638 PMC4270851 25351843

[4] Farrington K, Covic A, Nistor I et al (2017) Clinical practice guideline on management of older patients with chronic kidney disease stage 3b or higher (EGFR < 45 mL/min/1.73 m^2^): a summary document from the European Renal Best Practice Group. Nephrol Dial Transplant 32:9–16 28391313 10.1093/ndt/gfw411

[5] Schell JO, Patel UD, Steinhauser KE et al (2012) Discussions of the kidney disease trajectory by elderly patients and nephrologists: a qualitative study. Am J Kidney Dis 59:495–503. 10.1053/j.ajkd.2011.11.023 22221483 10.1053/j.ajkd.2011.11.023 PMC3626427

[6] Wachterman MW, Marcantonio ER, Davis RB et al (2013) Relationship between the prognostic expectations of seriously Ill patients undergoing hemodialysis and their nephrologists. JAMA Intern Med 173:1206–1214. 10.1001/jamainternmed.2013.6036 23712681 10.1001/jamainternmed.2013.6036 PMC3982381

[7] Noble H, Brazil K, Burns A et al (2017) Clinician views of patient decisional conflict when deciding between dialysis and conservative management: qualitative findings from the PAlliative Care in chronic Kidney diSease (PACKS) study. Palliat Med 31:921–931. 10.1177/0269216317704625 28417662 10.1177/0269216317704625

[8] Ghanem S, Hossri S, Fuca N et al (2020) Patient-nephrologist prognostic awareness and discordance in end stage renal disease on renal replacement therapy. Int Urol Nephrol 52:765–773. 10.1007/s11255-020-02420-2 32125588 10.1007/s11255-020-02420-2

[9] Frazier R, Levine S, Porteny T et al (2022) Shared decision making among older adults with advanced CKD. Am J Kidney Dis 80:599–609. 10.1053/j.ajkd.2022.02.017 35351579 10.1053/j.ajkd.2022.02.017

[10] Ouzzani M, Hammady H, Fedorowicz Z, Elmagarmid A (2016) Rayyan—a web and mobile app for systematic reviews. Syst Rev 5:210. 10.1186/s13643-016-0384-4 27919275 10.1186/s13643-016-0384-4 PMC5139140

[11] Cardinal H, Ballesteros Gallego F, Affdal A, Fortin MC (2020) Canadian transplant nephrologists’ perspectives on the decision-making process for accepting or refusing a kidney from a deceased organ donor. Clin Transplant. 10.1111/ctr.13793 31989699 10.1111/ctr.13793

[12] Finlay J, Wilson T, Javaheri PA et al (2020) Patient and physician perspectives on shared decision-making for coronary procedures in people with chronic kidney disease: a patient-oriented qualitative study. CMAJ Open 8:E860–E868. 10.9778/cmajo.20200039 33303572 10.9778/cmajo.20200039 PMC7867031

[13] Tuot DS, Crowley ST, Katz LA et al (2022) Usability testing of the kidney score platform to enhance communication about kidney disease in primary care settings: qualitative think-aloud study. JMIR Form Res. 10.2196/40001 36170008 10.2196/40001 PMC9557760

[14] Dowen F, Sidhu K, Broadbent E, Pilmore H (2017) Communicating projected survival with treatments for chronic kidney disease: patient comprehension and perspectives on visual aids. BMC Med Inform Decis Mak. 10.1186/s12911-017-0536-z 28934951 10.1186/s12911-017-0536-z PMC5607842

[15] Engels N, van der Nat PB, Ankersmid JW et al (2022) Development of an online patient decision aid for kidney failure treatment modality decisions. BMC Nephrol. 10.1186/s12882-022-02853-0 35794539 10.1186/s12882-022-02853-0 PMC9257566

[16] Ozdemir S, Choong LHL, Gan SWS et al (2021) Patient decision aid development for older adults with end-stage kidney disease in Singapore. Kidney Int Rep 6:2885–2896. 10.1016/j.ekir.2021.08.027 34805639 10.1016/j.ekir.2021.08.027 PMC8589693

[17] Patzer RE, McPherson L, Basu M et al (2018) Effect of the iChoose Kidney decision aid in improving knowledge about treatment options among transplant candidates: a randomized controlled trial. Am J Transplant 18:1954–1965. 10.1111/ajt.14693 29446209 10.1111/ajt.14693 PMC6510396

[18] Zimmermann CJ, Jhagroo RA, Wakeen M et al (2020) Opportunities to improve shared decision making in dialysis decisions for older adults with life-limiting kidney disease: a pilot study. J Palliat Med 23:627–634. 10.1089/jpm.2019.0340 31930929 10.1089/jpm.2019.0340 PMC7232635

[19] Damron KC, Friedman R, Inker LA et al (2022) Treating early-stage CKD with new medication therapies: results of a CKD patient survey informing the 2020 NKF-FDA scientific workshop on clinical trial considerations for developing treatments for early stages of common, chronic kidney diseases. Kidney Med 4:100442. 10.1016/j.xkme.2022.100442 35372821 10.1016/j.xkme.2022.100442 PMC8967726

[20] Peeters P, Van Biesen W, Veys N et al (2016) External validation of a risk stratification model to assist shared decision making for patients starting renal replacement therapy. BMC Nephrol. 10.1186/s12882-016-0253-3 27055653 10.1186/s12882-016-0253-3 PMC4823864

[21] Wilson TA, Hazlewood GS, Sajobi TT et al (2023) Preferences of patients with chronic kidney disease for invasive versus conservative treatment of acute coronary syndrome: a discrete choice experiment. J Am Heart Assoc. 10.1161/JAHA.122.028492 36892063 10.1161/JAHA.122.028492 PMC10111540

[22] Germain MJ (2015) How to integrate predictions in outcomes in planning clinical care. Blood Purific 39:65–69 10.1159/000368946 25661755

[23] Highet A, Cassidy DE, Gomez-Rexrode AE et al (2020) Introduction to the best-case/worst-case framework within transplantation surgery to improve decision-making for increased risk donor organ offers. Prog Transplant 30:368–371. 10.1177/1526924820958116 32959728 10.1177/1526924820958116

[24] Pencina MJ, Parikh CR, Kimmel PL et al (2019) Statistical methods for building better biomarkers of chronic kidney disease. Stat Med 38:1903–1917. 10.1002/sim.8091 30663113 10.1002/sim.8091

[25] Scherer JS, Swidler MA (2014) Decision-making in patients with cancer and kidney disease. Adv Chron Kidney Dis 21:72–80 10.1053/j.ackd.2013.07.005 24359989

[26] Winterbottom A, Stoves J, Ahmed S et al (2023) Patient information about living donor kidney transplantation across UK renal units: a critical review. J Ren Care 49:45–55. 10.1111/jorc.12404 34791808 10.1111/jorc.12404

[27] Cassidy BP, Getchell LE, Harwood L et al (2018) Barriers to education and shared decision making in the chronic kidney disease population: a narrative review. Can J Kidney Health Dis 5:205435811880332. 10.1177/2054358118803322 10.1177/2054358118803322 PMC6236635 30542621

[28] James LJ, Wong G, Tong A et al (2021) Discrete choice experiments to elicit patient preferences for decision making in transplantation. Transplantation 105:960–967. 10.1097/TP.0000000000003500 33093407 10.1097/TP.0000000000003500

[29] Winterbottom AE, Mooney A, Russon L et al (2021) Kidney disease pathways, options and decisions: an environmental scan of international patient decision AIDS. Nephrol Dial Transplant 35:2072–2082. 10.1093/NDT/GFAA102 10.1093/ndt/gfaa102 PMC7716808 32830240

[30] Anderson RT, Cleek H, Pajouhi AS et al (2019) Prediction of risk of death for patients starting dialysis: a systematic review and meta-analysis. Clin J Am Soc Nephrol 14:1213–1227. 10.2215/CJN.00050119 31362990 10.2215/CJN.00050119 PMC6682819

[31] Harwood L, Clark AM (2013) Understanding pre-dialysis modality decision-making: a meta-synthesis of qualitative studies. Int J Nurs Stud 50:109–120 22560169 10.1016/j.ijnurstu.2012.04.003

[32] Mak S, Thomas A (2022) Steps for conducting a scoping review. J Grad Med Educ 14:565–567. 10.4300/JGME-D-22-00621.1 36274762 10.4300/JGME-D-22-00621.1 PMC9580325

[33] Arksey H, O’Malley L (2005) Scoping studies: towards a methodological framework. Int J Soc Res Methodol 8:19–32. 10.1080/1364557032000119616

