# Supplementary material for: Communicating health risk in chronic kidney disease: a scoping review
Source: J Nephrol. 2024 Nov 8;38(1):101–10. doi: 10.1007/s40620-024-02098-0 (PMC11903627; doi:10.1007/s40620-024-02098-0)
Supplement: Supplementary file 1 — Supplementary file1 (PDF 141 kb) [file 40620_2024_2098_MOESM1_ESM.pdf]

Communicating health risk in chronic kidney disease: A scoping review

Emma Caton[1], Ros Aird[2], Maria Da Silva-Gane[3], Sivakumar Sridharan[1,3], David Wellsted[1], Shivani Sharma[4], Ken Farrington[1,3].

[1] School of Life and Medical Sciences, University of Hertfordshire, Hertfordshire, UK.
[2]Lister Area Kidney Patients Association, Stevenage, UK
[3]Department of Renal Medicine, Lister Hospital, East and North Hertfordshire NHS Trust, Stevenage, UK
[4]College of Business and Social Sciences, Aston University, Birmingham, UK

Corresponding Author: Emma Caton e.caton2@herts.ac.uk

Table S1. Search terms

**CONCEPT A: Kidney Disease & Treatment**	1. Kidney Disease<br>2. Renal Disease<br>3. Kidney Failure<br>4. Renal Failure<br>5. Dialysis<br>6. Haemodialysis<br>7. Hemodialysis<br>8. End Stage Kidney Disease<br>9. End Stage Renal Disease<br>10. Transplant<br>11. Transplantation<br>12. Disease Progression<br>13. Conservative Management<br>14. Conservative Treatment<br>15. Conservative Care<br>16. Palliative Care
**17. 1 OR 2 OR 3 OR 4 OR 5 OR 6 OR 7 OR 8 OR 9 OR 10 OR 11 OR 12 OR 13 OR 14 OR 15 OR 16**	
**CONCEPT B: Risk communication**	18. Risk Communication<br>19. Communicating Risk<br>20. Prognosis Communication<br>21. Communicating Prognosis<br>22. Shared Decision Making
**23. 18 OR 19 OR 20 OR 21 OR 22**	
**24. 17 AND 23**	
